# Colonic stenting as bridge to surgery versus emergency surgery for management of acute left-sided malignant colonic obstruction: a multicenter randomized trial (Stent-in 2 study)

**DOI:** 10.1186/1471-2482-7-12

**Published:** 2007-07-03

**Authors:** Jeanin E van Hooft, Willem A Bemelman, Ronald Breumelhof, Peter D Siersema, Philip M Kruyt, Klaas van der Linde, Roeland A Veenendaal, Marie-Louise Verhulst, Andreas W Marinelli, Josephus JGM Gerritsen, Anne-Marie van Berkel, Robin Timmer, Marina JAL Grubben, Pieter Scholten, Alfons AM Geraedts, Bas Oldenburg, Mirjam AG Sprangers, Patrick MM Bossuyt, Paul Fockens

**Affiliations:** 1 Department of Gastroenterology, Academic Medical Center, Amsterdam, The Netherlands; 2 Department of Surgery, Academic Medical Center, Amsterdam, The Netherlands; 3 Department of internal medicine, Diaconessenhuis Hospital, Utrecht, The Netherlands; 4 Department of Gastroenterology, Erasmus Medical Center, Rotterdam, The Netherlands; 5 Department of Surgery, Gelderse Vallei Hospital, Ede, The Netherlands; 6 Department of Gastroenterology, Medical Center Leeuwarden, Leeuwarden, The Netherlands; 7 Department of Gastroenterology, Leiden University Medical Center, Leiden, The Netherlands; 8 Department of Gastroenterology, Máxima Medical Center, Eindhoven, The Netherlands; 9 Department of Surgery, Medical Center Haaglanden, Den Haag, The Netherlands; 10 Department of Surgery, Medisch Spectrum Twente, Enschede, The Netherlands; 11 Department of Gastroenterology, Rode Kruis Hospital, Beverwijk, The Netherlands; 12 Department of Gastroenterology, St Antonius Hospital, Nieuwegein, The Netherlands; 13 Department of Gastroenterology, St Elisabeth Hospital, Tilburg, The Netherlands; 14 Department of Gastroenterology, St Lucas Andreas Hospital, Amsterdam, The Netherlands; 15 Department of Gastroenterology, Onze Lieve Vrouwe Gasthuis, Amsterdam, The Netherlands; 16 Department of Gastroenterology, University Medical Center, Utrecht, The Netherlands; 17 Department of Medical Psychology, Academic Medical Center, Amsterdam, The Netherlands; 18 Department of Clinical Epidemiology and Bio-statistics, Academic Medical Center, Amsterdam, The Netherlands

## Abstract

**Background:**

Acute left-sided colonic obstruction is most often caused by malignancy and the surgical treatment is associated with a high mortality and morbidity rate. Moreover, these operated patients end up with a temporary or permanent stoma. Initial insertion of an enteral stent to decompress the obstructed colon, allowing for surgery to be performed electively, is gaining popularity. In uncontrolled studies stent placement before elective surgery has been suggested to decrease mortality, morbidity and number of colostomies. However stent perforation can lead to peritoneal tumor spill, changing a potentially curable disease in an incurable one. Therefore it is of paramount importance to compare the outcomes of colonic stenting followed by elective surgery with emergency surgery for the management of acute left-sided malignant colonic obstruction in a randomized multicenter fashion.

**Methods/design:**

Patients with acute left-sided malignant colonic obstruction eligible for this study will be randomized to either emergency surgery (current standard treatment) or colonic stenting as bridge to elective surgery. Outcome measurements are effectiveness and costs of both strategies. Effectiveness will be evaluated in terms of quality of life, morbidity and mortality. Quality of life will be measured with standardized questionnaires (EORTC QLQ-C30, EORTC QLQ-CR38, EQ-5D and EQ-VAS). Morbidity is defined as every event leading to hospital admission or prolonging hospital stay. Mortality will be analyzed as total mortality as well as procedure-related mortality. The total costs of treatment will be evaluated by counting volumes and calculating unit prices. Including 120 patients on a 1:1 basis will have 80% power to detect an effect size of 0.5 on the EORTC QLQ-C30 global health scale, using a two group t-test with a 0.05 two-sided significance level. Differences in quality of life and morbidity will be analyzed using mixed-models repeated measures analysis of variance. Mortality will be compared using Kaplan-Meier curves and log-rank statistics.

**Discussion:**

The Stent-in 2 study is a randomized controlled multicenter trial that will provide evidence whether or not colonic stenting as bridge to surgery is to be performed in patients with acute left-sided colonic obstruction.

**Trial registration:**

Current Controlled Trials ISRCTN46462267.

## Background

Colorectal cancer is the second most common cancer in women and the third most common in men in the Netherlands [1]. The incidence in the Netherlands in 2003 was 9898 new cases, men and women were equally affected (Dutch Cancer Registry) [2]. Literature shows that between 7 and 29% of the patients present with a sub-total or total bowel obstruction [3,4].

Conventionally these patients are treated with emergency surgery to restore luminal patency. These emergency operations, involving an unprepared and obstructed bowel, include a variety of procedures ranging from a loop colostomy (blow-hole) to a Hartmann's procedure or even a subtotal colectomy. These interventions have a mortality rate of 15–34% and a morbidity rate of 32–64% despite advances in perioperative care [4-8]. It is well recognized that in a considerable number of these patients the ostomies will not be closed because of old age, metastatic disease or poor condition [3,7]. Patients with a permanent stoma do report a significant lower health-related quality of life than comparable patients without colostomy and require costly stoma material [9-12].

Since the early 1990s colonic stenting has been introduced, mainly in the left-sided colon, to restore luminal patency. It can be applied as a preoperative treatment to prepare patients for elective surgery as well as a definitive palliative procedure in patients with incurable disease [13]. In uncontrolled studies stent placement before elective surgery has been suggested to improve the patients' clinical condition, decreasing mortality, morbidity and number of colostomies [4,5,7,8]. Additionally this temporary procedure gives the opportunity to perform accurate tumor staging, leading to avoidance of surgery in patients with disseminated disease or unacceptable surgical risk [7,14-16]. In these patients the colonic stent may serve as permanent palliation. Colonic stenting also creates the opportunity to give neo-adjuvant therapy, improving patients' prognosis.

A systematic review by Sebastian et al. of 54 uncontrolled trials and case reports on placement of self-expandable metal stents revealed a technical success rate of 90–100%, a clinical success rate of 84–94% and clinical success when used as bridge to surgery of 71.7%. Major complications related to stent placement include perforation (4%), stent migration (11.8%) and re-obstruction (7.3%), causing a cumulative mortality of 0.58% [13]. It has to be underlined that stent perforation can lead to peritoneal tumor spill, changing a potentially curable disease in an incurable one. Minor complications like rectal bleeding (5%), transient anorectal pain (5%) and fecal impaction can mostly be managed conservatively [17,18].

The overall influence of colonic stenting on patient's quality of life is unknown. It is important to realize that stenting so far has mainly been done by experts and the published results are mostly uncontrolled, subjective to selection bias and do not include the learning curves.

To date a randomized comparison between the treatment with self-expandable stents before elective surgery and emergency surgery for malignant acute colorectal obstruction has not been published. We propose to compare these two treatment algorithms in a prospective multicenter randomized setting in terms of effectiveness (primary outcome measure) and healthcare costs.

## Methods/design

### Study objectives

To compare, in a prospective randomized fashion, colonic stenting followed by elective surgery with emergency surgery for the management of acute left-sided malignant colonic obstruction in terms of effectiveness. Effectiveness will be evaluated in terms of quality of life, morbidity and mortality.

### Study design

**Figure 1:**
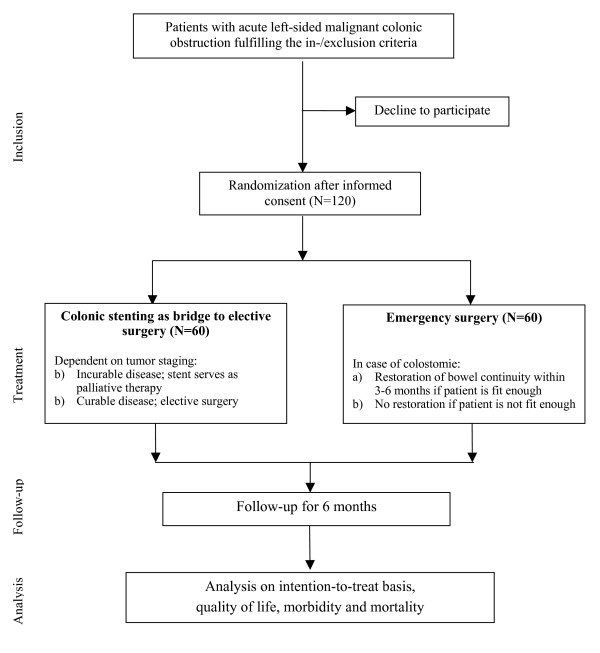
Flowchart Stent-in 2 study.

The Stent-in 2 study is a randomized multicenter trial with 4 academic and 11 regional hospitals participating in the project. Patients presenting with confirmed acute left-sided malignant colonic obstruction will be asked for informed consent if the inclusion and exclusion criteria are met. Computerized randomization will take place after informed consent has been obtained. Randomization will be performed centrally at the Academic Medical Center, using computer-generated lists prepared by the Department of Clinical Epidemiology and Bio-statistics. Lists will be constructed with randomly permuted blocks per stratum, in which strata will be defined by center (participating hospital). Patients will be randomized to either emergency surgery (current standard treatment) or colonic stenting as bridge to elective surgery (figure 1). Treatment will be initiated within 24 hours after randomization.

### Study population

The study population consists of patients with acute left-sided malignant colonic obstruction. Inclusion criteria are: symptoms of left-sided (colon descendens, sigmoid or rectum) malignant colonic obstruction existing less than one week, defined as obstructive symptoms with dilation of the colon on either plain abdominal X-ray and typical abnormalities on a gastrografin enema study or CT-abdomen with contrast compatible with a malignant colonic stricture; age above 18 years; signed informed consent. Exclusion criteria are: peritonitis, perforation, fever, sepsis or other serious complications demanding urgent surgery; American Society of Anesthesiologists (ASA) IV or V; obstruction due to non-colonic malignancies or from a benign origin: distal tumor margin less than 10 cm from the anal verge; incapability to complete self-report quality of life questionnaires.

### Outcome parameters

Effectiveness and costs of both strategies will be compared. Effectiveness will be evaluated in terms of quality of life, morbidity and mortality. Quality of life will be measured with the following standardized questionnaires: EORTC QLQ-C30 (European Organization for Research and Treatment of Cancer (EORTC) Quality of Life Questionnaire C30), the colorectal cancer specific EORTC QLQ-CR38 (European Organization for Research and Treatment of Cancer (EORTC) Quality of Life Questionnaire CR38), the EQ-5D (EuroQol 5 dimensions) and the EQ-VAS (EuroQol visual analogue scale). Morbidity is defined as every event leading to hospital admission or prolonging hospital stay. Mortality will be analyzed as total mortality as well as procedure-related mortality.

Cost items will include costs of hospital admissions and readmissions (operation, nursing days, outpatient visits), institutional care (nursing homes, hospice), home care, medication and other health care providers as well as direct non-medical costs (travel expenses).

### Participating centers

Fifteen Dutch hospitals including 4 academic and 11 non academic are currently participating in this trial. Specific requirement is to assure that enteral stent placement is performed by an experienced gastroenterologist. Gastroenterologists are considered experienced after placement of at least 20 enteral stents of which 10 colonic. In case no experienced gastroentorologist is available the patient should be referred to a nearby hospital with sufficient experience. The patient can always be referred for stent placement to the Academic Medical Center in Amsterdam. Laparoscopic sigmoidectomy or stoma creation is allowed if sufficient experience is available (> 20 cases).

### Ethics

This study is conducted in accordance with the principles of the Declaration of Helsinki and 'good clinical practice' guidelines. The protocol has been approved by the Medical Ethical Committee of the Academic Medical Center in Amsterdam and the local Ethical Committees of the participating centers. Prior to randomization informed consent will be obtained from all patients.

### Study outline

Patients presenting with confirmed acute left-sided malignant colonic obstruction will be asked for informed consent if the inclusion and exclusion criteria are met. Computerized randomization will take place after written informed consent has been obtained according to the Guidelines of Clinical Research in Humans. Randomization will be performed centrally at the Academic Medical Center, using computer-generated lists prepared by the Department of Clinical Epidemiology and Bio-statistics. Patients will be randomized to either emergency surgery or colonic stenting as bridge to elective surgery. Treatment will be initiated within 24 hours after randomization.

#### Colonic stenting

##### A) Material

For this study, the enteral stent which has been most frequently used in clinical studies will be employed (Enteral Wallstent™ (Boston Scientific, Natick, MA)) [13,18]. This through-the-scope stent is available in the length of 6 and 9 cm, with a diameter of 22 mm.

##### B) Procedure

After preparation of the distal colon with an enema, colonoscopy is performed. In all patients prophylactic antibiotics will be administered, covering aerobic and anaerobic bacteria. After endoscopic visualization of the lesion, biopsies will be taken and the length of the lesion will be measured (fluoroscopically). In case a normal colonoscope or sigmoidoscope can transverse the lesion there is no indication for stent placement and the patient will be considered a drop out. The obstructive lesion should not be dilated prior to stent placement. Stents will always be placed over a guidewire and under fluoroscopic guidance. A metallic, uncovered, self-expandable stent will be implanted, at least 3 cm longer (1,5 cm at both sides) than the lesion. If the stent does not cover the entire length of the tumor, a second overlapping stent will be placed. The correct position of the stent will be confirmed using fluoroscopy and endoscopy.

##### C) Post-procedure

Successful decompression is defined as restoration of intestinal transit based on resolution of obstructive symptoms on plain abdominal X-ray, production of stools and/or resolution of nausea and vomiting. If successful decompression is achieved and the condition of the patient is stabilized, tumor staging of the disease will be performed (CT-scan/plain chest X-ray) within one week. If any of the following conditions will be present, patients will be considered not suitable for surgical therapy and the self-expandable stent will serve as a palliative therapy: patients at high risk due to persistent comorbidity, advanced pelvic disease, peritoneal carcinomatosis and/or unresectable metastatic lesions. If decompression is not achieved within time frame of 3 days, or if clinically required, patients will be treated surgically but will not cross over to the surgical arm in the analysis.

##### D) Elective surgery after stenting

Candidates for elective surgery will be operated on after completion of the preoperative screening, preferably between day 5 and 14 after inclusion, at the latest four weeks after inclusion. If possible and convenient, patients will be discharged between diagnostic work-up and surgery. After preoperative bowel preparation and administration of antibiotics both according to the hospital protocol resection of the tumor will be performed, the extension of which will be determined by the specific characteristics of the malignant process. During surgical exploration, the position and efficacy of the stent will be evaluated.

#### Emergency surgery

##### A) Procedure

Patients will be operated according conventional standards within 24 hours after randomization. Whether a (palliative) resection or fecal deviation is done is at the discretion of the surgeon. Surgical options according to current standards are loop colostomy (blow-hole), resection with primary anastomosis with or without ileostomy, Hartmann's procedure, (sub)total colectomy with ileostomy or ileorectal anastomosis.

##### B) Post-procedure

After emergency surgery when the condition of the patient is stabilized, tumor staging of the disease will be performed (CT-scan/plain chest X-ray). In case of a colostomy an attempt at restoration of bowel continuity should preferable be performed within 3–6 months after inclusion. The colostomy will serve as a definitive solution if the patient refuses a re-operation or when a re-operation is judged to carry an unacceptable risk (ASA IV of V).

#### Surgical re-anastomosis

Candidates for surgical re-anastomosis will be operated on after completion of tumor staging and when considered stable. If possible and convenient, patients will be discharged between diagnostic work-up and re-anastomosis.

After preoperative bowel preparation and administration of antibiotics both according to the hospital protocol a re-anastomosis will be performed in accordance with conventional standards.

### Statistical analysis

#### Intention to treat

The analysis will be performed in accordance with the intention to treat principle.

#### Sample size calculation

Including 120 patients and randomizing them on a 1:1 basis will have 80% power to detect an effect size of 0.5 on the EORTC QLQ-C30 global health scale, using a two group t-test with a 0.05 two-sided significance level [19,20].

An effect size of 0.5 is chosen because a recent systematic review based on 38 studies, indicated a "remarkable universality" among estimates of clinical significance that centered around 0.5 effect size [21]. These authors recommend an effect size of 0.5 to serve as a default value for clinically significant change on quality of life measures used with chronic disease patients, when more specific information is missing, as is the case in patients with malignant colonic obstruction. Sloan et al. argue that a 0.5 effect size is even a conservative estimate that is likely to be clinically meaningful [22]. Based on these data we expect an effect size of 0.5 to be realistic in our group of patients.

### Data collection and monitoring

Patients will be followed for a period of 6 months. The questionnaires (EORTC QLQ-C30, EORTC QLQ-CR38, EQ-5D and EQ-VAS) are filled in by the patients on the day of randomization and on week 4, 12, and 24 after randomization. In addition the patients will be asked to hand out a small questionnaire on informal care to their caregivers on week 4, 12 and 24. The questionnaires will be sent to the patients by post, collection will be safeguarded by a trial nurse. The trial nurse will also follow the patients during their hospital stay after the intervention and contact them by telephone every two weeks to assess complications, re-interventions, re-admissions, visits to the outpatient clinic and missing items of the collected quality of life questionnaires. An electronic Case Record Form (CRF) will include general patients' data (sex, age, localization tumor, etc.), patients' responses to quality of life questionnaires (EORTC QLQ-C30, EORTC QLQ-CR38, EQ-5D and EQ-VAS), informal care questionnaires and data concerning type of intervention, complications, mortality, duration of hospital and intensive care stay. This electronical Case Record Form is based on the paper source filled out by the treating physician or the patient him-/herself and put into the data base by a trial nurse. The trial coordinator will monitor the data of every included patient.

### Data analysis

Differences in quality of life and morbidity will be analyzed using mixed-models repeated measures analysis of variance, accounting for differences in survival between groups. Mortality will be compared using Kaplan-Meier curves and log-rank statistics.

The primary analysis of the quality of life data will be performed using mixed-models analysis of variances for repeated measures. Missing data will be handled based on available data approach.

We will apply an extension of the Q-Twist method to estimate differences in quality-adjusted survival between groups.

A data and safety monitoring committee will safeguard trial continuation based on safety and effectiveness data. They will perform an interim analysis after 60 included patients have reached a one month follow-up.

### Economic evaluation

Health care costs include costs of hospital admissions and readmissions (operation, nursing days, outpatient visits), institutional care (nursing homes, hospice), home care, informal care, medication and other health care providers as well as direct non-medical costs (travel expenses). These costs will be estimated for the period after inclusion until 6 months. Data on hospital care will be collected from the hospital information systems and CRFs. The participating clinicians will fill out CRFs during control visits, re-interventions and admissions. Use of institutional care, home care and other health care providers will be collected with a short checklist, used by a special trained research nurse at 14 days interval until 6 months after inclusion. Informal care after discharge will be registered as time spent on informal care giving. This will be measured using the recall questionnaire as introduced by van den Berg et al[23]. Those questionnaires will be handed out by the patients to their caregivers on week 4, 12 and 24 after inclusion.

Real unit costs will be estimated for the surgical interventions and stent placement on the basis of resource use by detailed measurement of manpower and personnel time, equipment, materials, housing and overhead, following the microcosting method. Costs of inpatient days in hospital will be estimated as real costs per day using detailed information from the financial department of the Academic Medical Center. For all other health care and informal care, unit costs will be estimated following Dutch guidelines using fees and standardized costs if considered representative, and adapted if necessary.

Patient outcome analysis will be expressed by calculating the costs per quality adjusted life-years (QALYs). For this calculation the standardized questionnaire (EQ-5D) will be used as this questionnaire can be translated to utilities with values from the UK general population.

### Final analysis

If the hypothesis holds, a strategy of colonic stenting combined with elective surgery will lead to a better quality of life, less morbidity, mortality and costs compared with the standard strategy of emergency surgery. In that case the effectiveness and costs of both strategies will be reported. If the alternative strategy turns out to be more effective but more costly than the standard treatment, the costs per QALY will be calculated. In a sensitivity analysis, the impact of the statistical uncertainty of major parameters in the cost-effectiveness analysis will be examined. Since costs per patient are typically highly skewed, non-parametric bootstrap techniques will be used to derive a 95% confidence interval for the differences in mean and median costs.

## Discussion

The concept of colonic stenting as bridge to elective surgery in patients with an acute left-sided malignant colonic obstruction has been developed to reduce the morbidity, mortality and numbers of colostomies. Although some might consider stenting as bridge to surgery the preferred approach, solid evidence is lacking. A previously conducted randomized trial comparing surgery with stenting for incurable left-sided malignant colonic obstruction had to be stopped because of an unexpectedly high number of perforations in the stented group [24]. For this reason, the Stent-in 2 study is conceived to compare colonic stenting as bridge to elective surgery with emergency surgery (current standard treatment) for the management of acute left-sided malignant colonic obstruction. This multicenter randomized study will compare those two strategies with regard to effectiveness and costs. The aim is to provide evidence whether or not colonic stenting as bridge to surgery is to be performed in patients with acute left-sided colonic obstruction.

## Competing interests

The author(s) declare that they have no competing interests.

## Authors' contributions

JEvH drafted the manuscript. WAB and PF co-authored the writing of the manuscript. PF is the principal investigator. BO, MAGS, PMMB participated in the design of the study. The other authors are local investigators. All authors edited the manuscript and read and approved the final manuscript.

## Pre-publication history

The pre-publication history for this paper can be accessed here:


